# The effect of nature on creativity through mental imagery

**DOI:** 10.1371/journal.pone.0315141

**Published:** 2025-01-09

**Authors:** Aaron C. Drake, Fiza Hasan, Arianna Gibson, Julia W. Y. Kam

**Affiliations:** 1 Department of Psychology, University of Calgary, Calgary, Alberta, Canada; 2 Hotchkiss Brain Institute, University of Calgary, Calgary, Alberta, Canada; Education University of Hong Kong, HONG KONG

## Abstract

Immersion in nature has been linked to wide-ranging benefits on mental health and cognitive functions, from reducing stress to enhancing creativity. However, a walk in nature is not always feasible, and whether a proxy for nature immersion via a mental walk in nature can elicit the same benefits as a physical walk remains largely unknown. Accordingly, the current study utilized guided imagery to examine whether a mental walk in nature would improve creativity in general and when compared to a mental walk in an urban environment. We implemented a within-subjects design, wherein participants completed both mental walk conditions (in a nature and urban environment) at least five days apart in counterbalanced order on an online platform. During each session, participants (*N* = 97) completed two pre-walk tasks assessing convergent (measured by the Remote Associates Test) and divergent creative thinking (measured by the Alternate Uses Test), followed by a mental walk in either a nature or urban environment, then finally the identical two post-walk creativity tasks. After five days, they repeated the same procedure with a mental walk in the other environment. While comparisons of post-walk creativity scores between the nature and urban environment did not significantly differ from each other, the comparisons between the pre- and post-walk creativity scores revealed a significant improvement in convergent creative thinking in the nature environment condition, but not the urban environment condition. Our results suggest that taking a mental walk in nature can enhance at least one aspect of creativity, therefore providing preliminary evidence for the potential to access the creative benefits of mentally immersing ourselves in nature. These findings have important implications for those who wish to enjoy the benefits of nature but are unable to readily access nature physically.

## Introduction

Spending time in nature is beneficial in many different domains. These established benefits may explain the increased creation of natural parks within the heart of urban metropolises, as it is believed that these parks will introduce all the beneficial aspects of nature into an urban environment [1]. However, it appears that we are spending increasingly less time in nature [2], which may be due to the demands of the work force that keep individuals indoors [3–5], the increasing pervasiveness of technologies that keep our attention on our devices [6–8], or changes in temperatures and weather due to climate change [9, 10]. Given the challenges that many face in spending time outdoors in nature, one potential solution is to bring nature to them. Specifically, if a walk in nature is not feasible, then would taking a mental walk in nature elicit similar benefits as a physical walk?

Converging lines of research have demonstrated the benefits of nature immersion in various domains [11–14]. These benefits have been observed across different levels of immersion, which refers to how engaged one is with nature, ranging from lower levels such as viewing images of nature [15], to higher levels such as taking a walk in nature [16, 17]. In terms of mental health, several studies have assessed the impact of “Shinrin-yoku”, which is a Japanese term for contact with nature and being more in touch with forests and their atmosphere [11, 12, 18]. One group found that a day of exposure to nature by both walking and sitting within forested areas improved several physical and self-reported measures of stress compared to that of an urban environment [18]. In addition to reducing stress, a meta-analysis assessing the effect of exposure to nature on emotional well-being found that nature exposure leads to an increase in positive affect, as well as a smaller but consistent decrease in negative affect [13]. This effect has been established via different methods, from actual exposure to natural settings [16, 17], to laboratory-based simulated nature exposure using photographs [15] and virtual reality [19]. These findings suggest that the overall positive influence nature’s exposure has on our physical and emotional well-being is robust against different approaches to experiencing natural environments.

In terms of cognitive functions, the Attention Restoration Theory proposed by Kaplan [14] suggests that exposure and immersion in nature can also restore our attentional abilities. In everyday life, we rely heavily on voluntary or directed attention for extended periods of time, especially when performing our job or schoolwork. This can drain our ability to direct and control our attention, which is considered a finite resource that can be fatigued and must be replenished [20–22]. The Attention Restoration Theory suggests that exposure to nature engages another type of attention—specifically involuntary attention—which therefore allows our voluntary or directed attention to rest and restore [14]. In contrast, urban environments are full of external stimuli (e.g. car horns, advertisements, construction noise) that constantly draw our attention. Using our top-down processes, we must either attend to them or filter them out [23]. Since urban environments pose more direct threats to our safety through traffic or construction, this bombardment of stimuli can drain our directed attention even further. This contrasts with the natural environments that can replenish them. Therefore, a break from our work and urban environments to immerse ourselves in a natural environment may improve our ability to focus on work once we return through the restoration of directed attentional capacity. There is evidence of this in studies showing greater improvement on cognitive task performance after nature exposure compared to urban exposure [16, 23, 24].

Beyond physical and mental health improvements, another potential benefit of nature concerns an increase in one’s ability to be creative and produce novel thoughts or ideas. Creativity is generally defined as one’s ability to create original and useful ideas [25–27], suggesting that it is a skillset that can be improved or manipulated. Within this general definition, there are two specific aspects of creativity that can be measured: divergent and convergent thinking [28, 29]. Divergent thinking is the process in which we create a multitude of solutions to a given problem [29], such as brainstorming various ideas that could all act as potential solutions. In contrast, convergent thinking is the process in which we generate the single best solution to a problem requiring a search through a broad solution set [30], such as a marketing team evaluating ideas for a novel marketing strategy to improve profits and then selecting the best solution. Given that these are distinct but interconnected processes [31], it is important to assess both aspects of creativity when determining how nature may impact one’s ability to be creative.

Many studies have assessed the impact of nature on both types of creative thinking. For example, Atchley et al. [32] found that after a three-day hiking experience in nature, participants performed better on a common measure of convergent thinking than those who had not experienced the hike. Other studies have also demonstrated that a multi-day experience in nature led to an improvement in the same convergent thinking measure [33, 34]. Similar benefits of nature have been reported in divergent thinking. For instance, participants who simply viewed videos of natural environments performed better on divergent thinking tasks as compared to viewing videos of urban scenes [35]. Other studies have also demonstrated that nature can benefit creativity across a wide variety of methods that immerse one in nature, including viewing images of nature [36, 37] or simply having an indoor plant visible in a room as compared to a magazine rack [38]. In addition, Palanica et al. [35] found that viewing a 2-dimensional video of nature without audio was just as effective in improving creativity as physical exposure to an outdoor park environment. Together, these studies demonstrate that the benefits of nature are salient enough to persist across a wide variety of methods and levels of immersion, from viewing passively to physically being present in and interacting with nature.

One level of immersion that has not been the focus of creativity research is guided imagery. Guided imagery involves presenting instructions to an individual to help them visualize and imagine themselves in a certain scenario or environment through storytelling or metaphors [39]. These instructions facilitate the creation of mental imagery, which refers to representations of perception in the mind in the absence of a physical stimulus [40, 41], and may involve individuals being guided to dynamically move through an environment referred to as a mental walk. Guided imagery has been found to reduce stress [42, 43], reduce blood pressure [44], reduce anxiety [45] and promote relaxation [46], demonstrating its beneficial effects on one’s mental state. Most previous research on guided imagery has focused on a mindfulness-based approach, instructing participants to focus on and control their breathing and other bodily functions. In contrast, a nature-based approach focuses on describing the environment in detail, instructing participants to focus on their surroundings. While taking a nature-based approach to guided imagery has been done in previous studies to study its impact on mental well-being [43, 45], none have used this approach to examine its impact on creativity.

### Current study

While many different methods of nature exposure have been examined in relation to creativity, no studies to our knowledge have examined whether a simulated experience using guided imagery could elicit these same effects on creativity through taking a “mental walk” in nature. Given that many people may be restricted in their abilities to go out in nature due to physical impairments, geographic location, or changes in weather or climate, it would be beneficial for people to be able to experience nature mentally without the need for physical exposure. Thus, this current online study examined whether a guided imagery experience in a natural environment using audio clips would lead to an improvement in creativity, and whether the magnitude of that improvement would be greater than improvements following a guided imagery experience in an urban environment. We hypothesized that the experience of a mental walk through a natural environment would lead to improvements in both convergent and divergent thinking. Moreover, the improvement in creative thinking following a walk in a natural environment would be greater than the improvement following a mental walk in an urban environment.

## Method

### Participants

One hundred and one university students from a Canadian university were recruited to participate in this study through the university’s internal research participation system. This sample size was determined by a power analysis based on previous studies of similar design that utilized physical and virtual immersion in nature. These studies reported effect sizes between 0.54 [36] and 0.86 [32]. Given that the current design is novel in terms of the modality of the ‘walk’ (via guided imagery), we adopted a more conservative estimate and aimed to detect a small-to-medium Cohen’s *d* effect size of 0.3. We used G*Power 3.1 [47] to conduct the power analysis, using the *t* tests: matched pairs, setting power to .80, and alpha level to .05. Our power analysis revealed that a sample of 90 participants would be required to detect the targeted effect size, and we recruited around 100 participants to account for a 10% attrition rate (given the two-part nature of this study) and potential missing data. Participants received course credit for participation. The mean age of the participants recruited was 19.57 years (*SD* = 2.85, range = 17 to 41). Of the 101 participants recruited, 86 identified as female, 14 as male, and one as non-binary; 89 were right-handed and 12 were left-handed. All participants were undergraduate students (*n* = 93 within fourth year of post-secondary education; *n* = 8 within fifth to seventh year of post-secondary education). Participants were excluded from subsequent analyses if they did not complete both parts of the study, if they spent longer than one and a half hours on either part of the study, or if they reported mind-wandering during the entirety of the mental imagery task. This resulted in four participants being excluded from the final analyses, resulting in a final sample of 97. The study was approved by the institutional Research Ethics Board (REB20-1433), and all participants indicated informed consent prior to participating. Recruitment for this study began on December 1, 2020, and concluded on March 10, 2021.

### Materials

#### Guided imagery audio

The guided imagery audio files were created using a combination of two existing guided imagery scripts for both the natural and urban conditions [43, 45]. The guided imagery scripts can be found in S1 File. Both audio files were approximately 10 minutes in length and were matched in duration, structure, and content to create balanced experiences across conditions (e.g., “The air is warm and comfortable. Sun filters through the trees, making a moving dappled pattern on the ground before you” for the natural condition; “The air is warm and comfortable. The sun shines around the tall buildings, making a steady pattern of shading on the ground before you” for the urban condition). It should be noted that within mental imagery, there are more specific types such as motor imagery. Motor imagery is a conscious process where an individual mentally simulates an action without physically performing it, such as visualizing oneself returning a tennis serve [48]. Notably, although our task instructs participants to mentally walk through an environment, our primary aim was to facilitate mental imagery broadly with the primary focus on the external environments themselves, with little focus on the movement. Therefore, we instructed participants to visualize the environment surrounding them through their sense of sight, touch, smell, and sound, with little explicit instructions on how to move their bodies. Additional sounds typically found in each environment were included throughout the entire guided imagery to increase immersion [49]. These consisted of birds chirping, running stream sounds, and sounds of wind for the natural condition, and sounds of traffic, construction, and passing pedestrians in the urban condition.

#### Remote Associates Test (RAT)

All participants completed 20 RAT questions both before and after exposure to the two mental walk conditions; therefore, they completed the RAT four times involving a different set of questions each time. The RAT assesses convergent thinking; it involves the presentation of three words, and the participant must think of a fourth word that connects to all three words in a similar way [50]. For example, if presented with the words “flag, vault, fishing,” the correct fourth word would be “pole” since it connects to all three in a similar way: “flagpole,” “pole vault,” and “fishing pole.” Four versions of the RAT were created, balanced in difficulty using data reported by Bowden & Jung-Beeman [51], with roughly equal numbers of questions of each difficulty level, resulting in 80 questions in total. Easy level questions were defined as items with a 70% or above success rate for the 15 second time limit, medium level questions were defined as those with a 31%–69% success rate, and difficult level questions were items with a 30% or less success rate. Participants were given 30 seconds for each RAT item, after which the screen advanced and recorded whatever response was provided in the text box. All questions included were compound RAT problems; therefore, all questions included words that could be combined into compound words rather than related through semantics or synonymy [52]. The purpose of enforcing consistency in the type of questions used in our study was to reduce confusion in performing the task. Fourteen questions were modified slightly from the original version due to regional differences in the use of terminology. For the RAT practice session, we extracted three questions, one at each of the easy, medium, and difficult levels from an online database, and were different from the ones used at testing [53].

#### Alternate Uses Test (AUT)

All participants completed a 3-item AUT before and after exposure to the mental walk conditions. The AUT assesses divergent thinking; it involves the presentation of a common item, such as a brick, and participants are asked to provide as many uncommon, unusual, and useful uses for this item as possible. Four versions of the AUT were created, with items sorted so as to not have multiple items in the same test with similar uses. This resulted in 12 AUT items in total (i.e., brick, car tire, pencil, paperclip, knife, clothes hanger, barrel, tin can, fishing net, spoon, washcloth, and broom). Participants were given two minutes for each AUT item, after which the screen advanced and recorded whatever response was provided in the text box. Given the relatively large pool of items necessary for presenting non-overlapping items across assessments and conditions within individuals, our AUT items were pulled from various sources [54–57]. Participants were presented with one practice item (bucket) with an example of a list of possible responses to familiarize them to the task. To quantify responses on the AUT, both the creativity of responses (originality), as well as the number of uses provided (fluency) were scored. For the originality measure, two independent raters scored responses on a scale of 0–3, with 0 being not creative at all (the intended use), and 3 being highly creative. To determine the level of agreement between their scores, Cohen’s kappa coefficient was calculated. This resulted in a kappa of .93, which is considered near-perfect agreement [58]. For the fluency measure, only unique, unrepeated uses of the item were counted and totaled by another independent rater. As independent raters are commonly used to quantify responses on the AUT [56, 57, 59, 60], we adopted the same approach to facilitate comparisons between our study and existing ones. Nonetheless, given potential concerns about the subjectivity of raters [61], we also implemented an AI-powered scoring method utilizing large language models [62]. The patterns of significance in our results did not change, suggesting both approaches to quantifying AUT responses led to the same overall results. Given the results did not change and this AI-powered scoring method was implemented post-hoc, we report details about this approach and its corresponding results in S1 File.

#### Perceived Restorativeness Scale

Participants completed a shortened version of the Perceived Restorativeness Scale consisting of 5 items after each mental walk [20]. This scale assesses the perceived restorativeness of the environment they experienced (e.g., “It was a place that was away from everyday demands and where I would be able to relax and think about what interests me”). It is based on Kaplan’s [14] four facets of Attention Restoration Theory: being away, extent, fascination, and compatibility. Participants responded to the question “How much does this statement apply to the place I was imagining during my mental walk?” measured on a 5-point Likert scale (1 = “Not at All” to 5 = “Completely”).

#### Four Factor Imagination Scale

Participants completed a shortened version of the Four Factor Imagination Scale [63]. The scale comprises 26-items that assess individual differences in imagination abilities and was included to account for imaginative differences between participants in a control analysis. Although the scale comprises of several factors (such as frequency, emotional valence, and directedness), our analysis focused on the complexity factor as this most relates to their ability to imagine the mental walk (e.g., “Most people seem to have more complex imaginations than me”). Participants were asked to indicate “How accurately or inaccurately the statements describe [their] normal daily experience” on a 6-point Likert scale (1 = “Very Inaccurate” to 6 = “Very Accurate”).

#### Nature Relatedness Scale—Experience Subscale

Participants completed the Experience Subscale of the Nature Relatedness Scale [64]. This 6-item subscale assesses the perceived connection between an individual and nature and was included to account for individual differences in their perceived connection to nature in a control analysis (e.g., “My ideal vacation spot would be a remote, wilderness area”). Participants were asked to “Rate the following items on how well they describe you” on a 5-point Likert scale (1 = “Strongly Disagree” to 5 = “Strongly Agree”).

#### Imagination Manipulation Check

A set of questions was designed to assess the extent to which participants ‘imagined’ the mental walk. The questions included measures to determine the level of detail present in participants’ mental walk, how much of it was in the first-person perspective, how often they mind-wandered, their mental fatigue, and their positive and negative feelings (e.g., “Did you have a specific location in mind while imagining yourself taking this walk?”). Participants responded to these questions on a 5-point Likert scale following each mental walk. This list of questions and their results are presented in S1 File.

#### Control questions

We asked participants to complete three control questions at the end of the study to assess their experience with using mindfulness/meditation practices, on 5-point Likert scale (1 = “None at All” to 5 = “Very Much”). In addition, two yes or no questions that asked whether they considered natural environments or urban environments to be beneficial to creativity were included to account for individual differences in perceived benefits of a given environment on creativity in control analyses.

### Procedure

Participants completed the study via the online survey platform Gorilla. There were two parts to the study, completed approximately five days apart. Each part took participants approximately one hour to complete, thus totaling two hours to complete the entire study. Participants received one course credit for each section of the study, for a maximum of two credits for full participation.

In part one of the study, participants first provided informed consent. They were then presented with an audio check question, which ensured that all participants had fully functional audio, as the guided imagery audio clip was essential to participation. Participants then answered several demographics questions, after which they were randomly assigned into the "Nature First" condition, or the "Urban First" condition using the randomization feature on Gorilla. The order of condition was counterbalanced across participants. Afterwards, participants were presented a practice session for the RAT assessing convergent thinking that contained three practice questions, followed by the pre-walk version of the RAT. Each version of the RAT was randomly assigned by Gorilla from the four possible options as described above. Then, participants were presented with the example item and responses as practice for the AUT assessing divergent thinking, followed by the pre-walk version of the AUT. Each version of the AUT was also randomly assigned by Gorilla from the four possible options as described above.

Next, participants were instructed to listen to the guided imagery audio, in which they were guided through a mental walk in either a natural or urban environment first. Following the mental walk, they were asked to complete the Imagination Manipulation Check and Perceived Restorativeness Scale to capture their experience in the imagined walk’s environment. Participants then completed the post-walk version of the RAT, followed by the post-walk AUT (with the versions of RAT and AUT randomly assigned by Gorilla). Following this, they completed the Four Factor Imagination Scale to capture their natural abilities at using their imagination.

After five days, participants were asked to complete part two of the study. They first completed another set of pre-walk RAT and AUT questions. Participants then went through the other version of the mental walk not previously experienced (natural or urban), followed by the completion of the Imagination Manipulation Check and Perceived Restorativeness Scale again. Participants then completed the final post-walk RAT and AUT questions, which were followed by the Nature Relatedness Scale—Experience Subscale and the set of control questions. This within-subjects design is more statistically powerful (as it removes individual variation) than a between-subjects design.

### Analysis plan

All statistical analyses were carried out using SPSS Statistics (v. 27). First, in order to examine whether a mental walk through a given environment would lead to improvements in creativity, we compared the extent of improvement in creativity before and after a mental walk within each condition. Specifically, we planned an exploratory analysis that involves separate two-tailed paired-samples *t*-tests comparing the pre- and post-walk creativity scores for the nature mental walk condition and the urban condition. For each condition, we implemented three separate *t*-tests for each creativity measure to determine if there was a post-walk relative to pre-walk improvement in RAT scores assessing convergent thinking, and originality and fluency scores on the AUT assessing divergent thinking.

Next, to address whether a mental walk through a natural environment would lead to greater improvements in creativity compared to an urban environment, our analysis involved comparing the post-walk creativity scores between the two conditions of mental walk environment. We planned to test this via a one-way repeated measures ANCOVA with environment (natural vs. urban) as the independent variable, the pre-walk creativity scores entered as a covariate, and post-walk creativity scores entered as dependent variables. Studies have shown that ANCOVA is the preferred analysis approach for pre-post studies when participants are randomly assigned to conditions [65] and has more power than a typical ANOVA approach in this scenario [66]. Thus, we planned three separate ANCOVAs for each dependent variable: one for the RAT scores, one for the originality score on the AUT, and one for the fluency score on the AUT. We also had planned to control for imaginative abilities by entering mean scores on the complexity factor of the Four Factor Imagination Scale and connectedness to nature using mean scores on the Nature Relatedness Scale—Experience Subscale entered as covariates in the same ANCOVA models. In summary, while the analysis that addresses the first hypothesis reveals whether or not a mental walk in nature would lead to improvements in creativity, this analysis allowed us to directly compare whether changes in pre- and post-walk creativity scores are greater in the natural walk condition compared to the urban walk condition.

We planned four control analyses to ensure any conditional differences could not be explained by alternative reasons. First, to examine whether there were any ordering effects in which participants imagined the mental walk environments, we included the order of conditions as a variable in the ANCOVA model described above. Second, we examined whether one’s imagination abilities impacted their creativity scores by comparing individuals with low and high imaginative abilities via a median split on the complexity factor of the Four Factor Imagination Scale. This would be examined by averaging participants’ scores on the FFIS to get a single score (range: 1–6), then splitting this on the median score (*Mdn* = 4.0) into low and high scores, excluding the median from analyses. We then implemented the same ANCOVA model as above, with low and high imaginative abilities entered as a between-subjects variable. Third, we planned a correlation analysis between mean scores on the Perceived Restorativeness Scale and post-walk creativity scores to determine whether participants who perceived their imagined environments as restorative scored higher on the creativity measures. Lastly, we planned to compare those who believe and do not believe natural and urban environments to be beneficial to creativity to determine whether personal beliefs about these environments impacted creativity. This was implemented utilizing the same ANCOVA model as above with the two belief groups entered as a between-subjects variable, with one analysis for each environment. These analyses were pre-registered on the Open Science Framework (https://osf.io/uachj).

## Results

Given the RAT and AUT scores are continuous variables, we tested the assumption of normality of these variables by examining the Shapiro Wilk test, skewness/kurtosis, and histograms of distributions. Although several Shapiro Wilk tests were significant (all *p* = .003 to .370), the kurtosis/skewness scores were all within the normal range and the histograms all appeared normally distributed. Information regarding all assumptions checking is presented in S1 File. Given most of these tests point to a normal distribution and combined with the robustness of ANOVAs to violations of normality, we used a parametric test to statistically address our objectives [67]. All subsequent analyses were compared to a critical alpha of .05 for statistical significance, and all confidence intervals presented are set to 95%.

**Fig 1:**
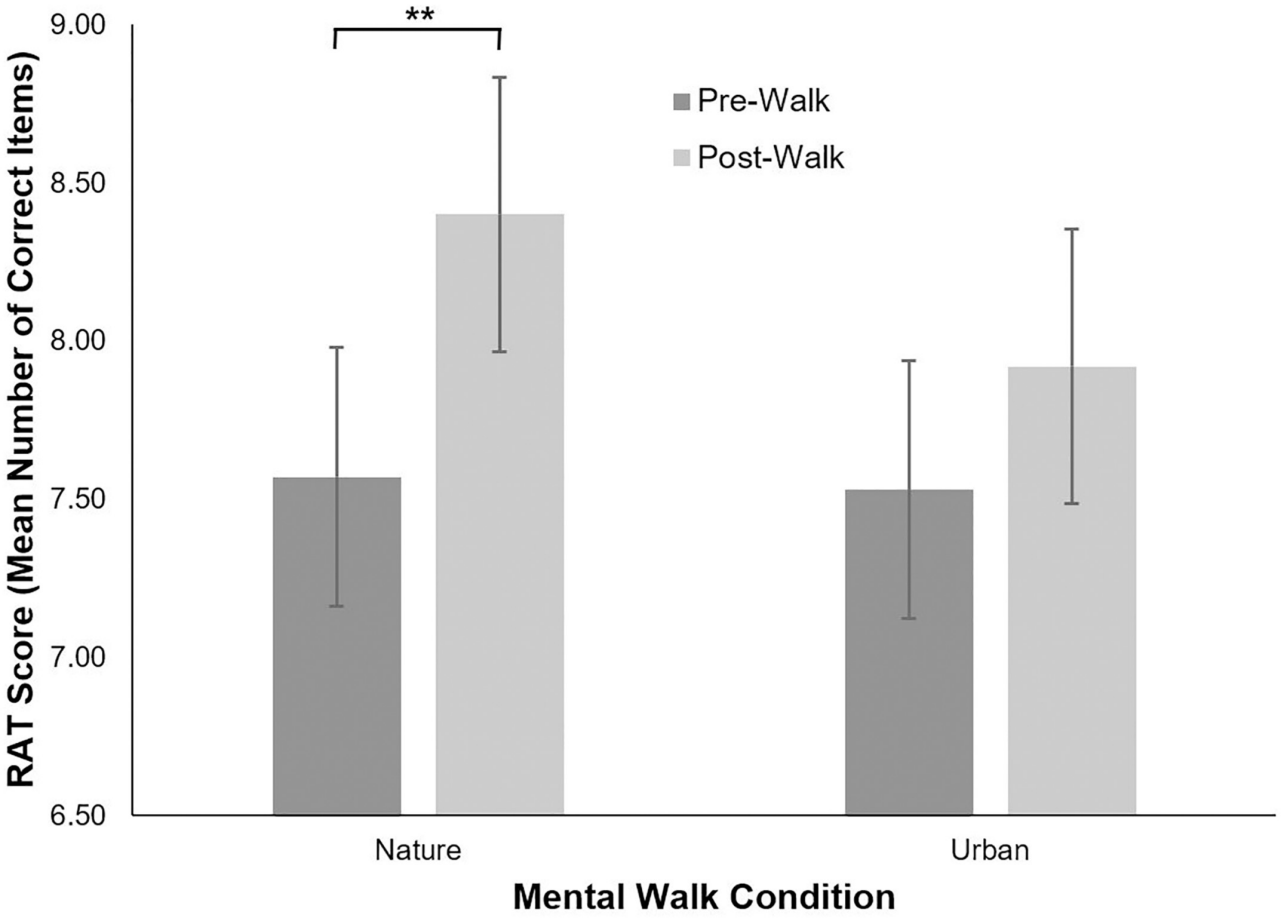
Mean Remote Associates Test (RAT) scores assessing convergent thinking before (pre) and after (post) a nature or urban mental walk using guided imagery. RAT scores in the nature mental walk condition significantly increased from pre- to post-walk timepoints, while scores in the urban condition did not. Error bars represent +/− 1 standard error of the mean. ** *p* < .01.

To examine whether each mental walk environment condition improved participants’ creativity scores from pre- to post-walk, we conducted separate paired-samples *t*-tests to compare the pre- and post-walk creativity scores for both the natural and urban conditions. Results for these analyses are reported in Table 1. For the RAT assessing convergent thinking, our results showed that for the natural environment, post-walk RAT scores were significantly higher than pre-walk RAT scores; difference between conditions (*ΔM*) = 0.84, 95% CI [1.43, 0.24]. In contrast, for the urban environment, post-walk RAT scores did not significantly differ from pre-walk RAT scores; *ΔM* = 0.39 [0.98, -0.19]. Thus, aligned with our first hypothesis, these analyses demonstrate that only a mental walk in a natural environment resulted in an increase in RAT scores from pre- to post-walk timepoints. The pre- and post-walk RAT scores for both mental walk conditions are shown in Fig 1.

**Table 1:** Descriptive and statistical results comparing pre- to post-walk creativity scores.

Task	Environment	Pre-Walk Scores		Post-Walk Scores		*t*	*p*	*d*
		*M*	*SD*	*M*	*SD*			
RAT	Nature	7.57	3.96	8.40	4.29	2.78	.007	0.28
	Urban	7.53	4.08	7.92	4.25	1.33	.186	0.14
AUT Originality	Nature	1.37	0.19	1.35	0.17	0.52	.603	0.05
	Urban	1.37	0.19	1.35	0.20	0.69	.491	0.07
AUT Fluency	Nature	16.82	6.65	16.94	7.26	0.26	.794	0.03
	Urban	16.25	5.86	16.05	6.66	0.45	.655	0.05

RAT = Remote Associates Test, AUT = Alternate Uses Test, *d* = Cohen’s *d*. *df* = 96 for all comparisons.

In order to examine whether this difference was shown with divergent thinking as assessed by the AUT, we also performed paired samples t-tests for both originality and fluency scores. For originality scores in the nature environment, there were no significant differences from pre-walk to post-walk; *ΔM* = 0.01 [-0.04, 0.06]. The same was true for originality scores in the urban environment from pre-walk to post-walk; *ΔM* = 0.02 [-0.03, 0.06]. For fluency scores in the nature environment, no significant differences were found between pre-walk and post-walk; *ΔM* = 0.11 [0.97, -0.74]. The same was true for the urban environment from pre-walk to post-walk; *ΔM* = 0.20 [-0.67, 1.06]. These findings suggest neither a mental walk in a natural or urban environment improved divergent thinking.

To examine whether the two environment conditions (natural and urban) differentially impacted participants’ creativity scores at the post-walk timepoint while controlling for their pre-walk scores, a repeated-measures ANCOVA was performed that included the pre-walk scores as a covariate, the mental walk condition as the within-subjects variable, and post-walk scores as the dependent variable. Results from these analyses are presented in Table 2. For all measures of convergent and divergent thinking assessed by the RAT and AUT respectively, the results showed that there were no significant differences between the post-nature and post-urban walk conditions. Contrary to our second hypothesis, these results indicate that improvements in creativity did not differ following a mental walk in a natural versus an urban environment after controlling for pre-walk levels of creative thought.

**Table 2:** ANCOVA results comparing post-walk creativity scores while controlling for pre-walk scores.

Task	Post-Nature Walk Scores		Post-Urban Walk Scores		*F*	*p*	*η*_*p*_^*2*^
	*M*	*SE*	*M*	*SE*			
RAT	8.40 [7.87, 8.93]	0.27	7.92 [7.38, 8.46]	0.27	0.01	.944	.00
AUT Originality	1.35 [1.32, 1.39]	0.02	1.35 [1.31, 1.39]	0.02	2.69	.104	.03
AUT Fluency	16.94 [16.08, 17.79]	0.43	16.05 [15.27, 16.84]	0.39	1.42	.236	.02

M = estimated marginal means, RAT = Remote Associates Test, AUT = Alternate Uses Test, *η*_*p*_^*2*^ = Partial Eta Squared. Brackets represent 95% confidence intervals. *df* = 1, 94 for all comparisons.

All four control analyses were conducted as planned. In the first control analysis, no ordering effects were found, suggesting whichever mental walk participants completed first did not differentially impact their performance on creativity scores. Secondly, there was no effect of participants’ ability to create mental imagery on their creativity scores. Thirdly, participants’ perception of how restorative their mental walk was had no impact on creativity scores. Lastly, participants’ beliefs about the benefits of urban and natural environments for creativity had no impact on their creativity scores. All these analyses are reported in S1 File.

## Discussion

The primary purpose of this study was to examine whether a mental walk in a natural environment using guided imagery could elicit similar benefits to creativity as demonstrated in real nature exposure. The results of this study partially support our hypotheses and suggest a benefit in convergent thinking following a mental walk in nature, but not following a mental walk in urban environments. In contrast to our second hypothesis, the post-walk performance levels on creativity tasks did not differ between a mental walk in nature versus urban environments. Therefore, our results provide partial evidence for the creative benefits of experiencing nature through internally oriented methods such as guided imagery. This finding is particularly important for individuals who are not able to physically immerse themselves in nature and has important theoretical and practical implications detailed below.

We found an improvement in convergent thinking following the nature mental walk, but not the urban mental walk condition. This was shown by an increase in the post-walk creativity scores relative to the pre-walk creativity scores on the RAT, but not when comparing post-walk scores across environments. This suggests that a mental walk in nature improved convergent thinking, but not to a greater extent than a walk in the city. These benefits of a nature walk on convergent thinking could not be attributed to participants’ ability to create mental imagery, nor their perception of the restorativeness of their mental walks. We further discuss demand characteristics and how they are unlikely to explain our findings in S1 File. Supporting one of our hypotheses, this finding is in line with previous research showing that exposure to nature across various levels of immersion can improve convergent thinking [32–34]. This adds to the growing evidence that being in nature, either physically or mentally, provides us with a restorative environment that allows us to replenish some of our directed attentional resources. From a theoretical standpoint, our finding demonstrates the benefits of nature according to the Attention Restoration Theory may also extend into the creativity domain. From a practical standpoint, these results provide preliminary evidence that the use of a guided imagery audio experience in nature might also elicit a similar benefit to one aspect of creativity as other types of nature exposure. This finding is relevant to employers and employees alike in industries in which creativity is core to the successful performance in their jobs, including science and fine arts among others.

Although a mental walk in nature improved scores of convergent thinking, this was not the case for divergent thinking. A potential explanation for these differential impacts is the timing of nature exposure interacting with the different mechanisms involved with both aspects of creativity. Convergent thinking, a process where one must come up with a single correct answer, is seen as involving more directed attention processes [68]. On the other hand, divergent thinking involves less constrained attention and more wandering of thoughts, facilitating the connection between many new and unrelated ideas. Given that nature is proposed to benefit convergent thinking through replenishing directed attention *after* the nature exposure, this effect should have lasting effects on convergent thinking [69]. Conversely, nature is proposed to benefit divergent thinking *during* the nature exposure, allowing one to think more broadly and develop new ideas while being immersed and experiencing soft fascination. Therefore, since we assessed creative thinking after the mental walk, this may benefit convergent thinking through replenished directed attention, and may not benefit divergent thinking to the same extent given assessment occurred after the walk. A future direction could selectively test the benefits of a mental walk in nature on divergent thinking during the exposure itself.

While this significant improvement in convergent thinking scores for the natural mental walk was observed, the amount of improvement in convergent thinking did not differ between a nature and urban walk. This was contrary to what we hypothesized, as we expected to find that a nature mental walk would result in greater benefits to creativity compared to the urban mental walk. While one could argue that the mental walk experience did not elicit a strong enough effect to detect differences between the conditions, it could also be that our participants found taking a mental walk using guided imagery was relaxing enough to elicit a similar change in post-walk convergent thinking scores across both conditions. There is some evidence for this in that post-walk RAT scores for the urban condition did numerically increase in relation to the pre-walk scores, but not significantly. Additional evidence for this was found in a study assessing the impact of a mental walk using guided imagery, which reported similar improvements in self-reported stress and mood for both natural and urban environments [43]. Thus, it may be possible that a mental walk in general could be beneficial for convergent thinking to varying degrees. Therefore, the natural and urban mental environments as guided by audio clips here may not have differed enough to elicit a differential benefit on creativity.

One reason for this may be due to the method of presentation. Given that both the nature and urban guided imagery audios were designed to be similar in structure, and both had to be presented at a comfortable audio level, the urban experience may not have been accurate enough to a real experience to diminish their attentional resources. In a real urban experience, sounds and visual stimuli are much more pervasive and less pleasant, and convey more real-life threats (e.g. the sounds of honking may be an informative cue to step away or walk faster to prevent a life-threatening accident). In order to rule out alternative explanations for any condition differences in creativity measures, the two audio files were created to be very similar to elicit comparable experiences. Additionally, the audio in the current study had to be presented at a safe volume, meaning the guided imagery audio was not nearly as intrusive as what a real urban experience would be, and it would also not represent actual threats in their immediate surrounding. Thus, the audio experience provided here may not have effectively created as disruptive of audio cues as a real-world urban experience that often drains attentional resources, and therefore resulted in a less contrasting mental experience than anticipated.

### Limitations

One limitation of this study is the possibility of learning effects, since every participant completed the creativity measure four times in total. It could be argued that the increase from pre- to post-walk RAT scores in the nature mental walk condition can be explained by this. However, this explanation is unlikely considering that participants were counterbalanced in the ordering of their conditions. Thus, only half received the natural condition first, and the other half received the urban first. If there was a large difference between pre- and post-walk RAT scores simply due to learning effects, then a significant improvement in creativity scores should have also been observed in the urban condition, which was not the case. In addition, our analysis of ordering effects was not significant, providing further evidence that this is an unlikely explanation. For example, if a learning effect was more prevalent for one condition over the other, those who received the urban walk first and the nature walk second would be expected to have higher scores following their nature walk compared to those in the opposite order. However, our analysis examining ordering effects tested this directly and showed that there was no difference in creativity scores regardless of which mental walk condition participants began with.

Another point of consideration is that the use of guided imagery may limit the coexistence of nature exposure and mind-wandering. Williams et al. [68] have proposed that nature benefits creativity through a gentle shift between external attention via soft fascination and internal attention via mind-wandering. In our study, participants may not have been able to freely experience these soft fascinations via mind-wandering during the guided imagery (as they were instructed to focus on imagining a natural environment), and therefore may not have been able to benefit from nature exposure in their creative responses to the same extent as this theory would predict. However, since the guided imagery was presented through an auditory modality, participants may still have had the freedom to wax and wane between being immersed in the environment and mind-wandering. There is evidence of this in participants’ reported mind-wandering levels during the guided imagery. As reported in S2 Table of S1 File, the majority of participants reported some levels of mind wandering during the mental imagery (96 of 99 in the nature condition and 95 of 99 in the urban condition). Thus, it appears that participants were able to mind wander while also creating mental imagery.

Furthermore, it is possible that not all participants actively participated in the guided imagery task. Given that participants completed this study online due to data collection constraints from COVID-19, we were unable to actively control the environment wherein they completed the study. Importantly, we implemented several measures to reduce this potential issue. First, we informed the participants about the requirements of the task, including a quiet environment and the duration of the study so they could plan accordingly. Second, we included an audio test to verify that participants had functioning speakers or headphones to ensure they could hear the guided imagery audio clips. Third, we excluded participants that took longer than an hour and a half to complete either session, which would suggest they were not paying attention and left the task running in the background. Fourth, we asked participants to rate the extent to which their mind wandered during each mental walk, which allowed us to exclude those who did not pay attention to the mental walk at all. Nevertheless, the possibility remains that some participants may not have paid full attention to the audio or have had distractions present that could have disrupted the immersion. However, it is unlikely that no participants attended to the audio, since we did observe systematic differences in their creativity performance before and after the nature mental walk audio.

Lastly, our results should be considered with the following issues of generalizability. Given that our sample was predominantly women (85%), our findings may show limited generalizability to both men and women. Because there were far fewer men (*n* = 14) in our sample, comparison analyses were not feasible, so these findings should be interpreted with caution in regard to their generalizability to men. However, it should be noted that research in creativity suggests that there are no notable differences in creative performance between male and female participants [70, 71], and those that do are inconsistent [72, 73]. Thus, it is unlikely that gender would play a significant role in our findings. Additionally, research has shown that performance on the RAT and AUT may not accurately translate to real-world creativity [31, 74]. While these tests aim to capture convergent and divergent thinking, which are important components of creativity, they may not translate to creative performance in the real world. Therefore, the results of this study should be considered tentatively in terms of their generalizability to real-world creativity.

### Future research

To extend these findings, future studies could examine the possibility of presenting these guided imagery mental walks during specific phases in the creative process. Of particular interest for future studies, the creative phase of incubation involves when the conscious process of creative thought ends, and one continues thinking about the problem subconsciously [75–78]. Studies have demonstrated benefits to creativity while in the incubation phase by inducing mind-wandering [79, 80]. Some studies have also reported that nature exposure is particularly beneficial during the incubation phase [69]. Thus, it could be possible that to see a stronger benefit to creativity, one would need to be exposed to a problem, become stuck in the incubation phase, and then be immersed within nature to assist in creating responses, subsequently advancing to the next phase of the creative process. In other words, to reap maximal benefits of nature, participants could be presented with convergent or divergent tasks that require a longer time to solve, then exposed to nature, and then re-introduced to the same items. For example, for the RAT, those items that were not solved or were incorrectly solved could be re-presented to determine whether a natural mental walk produces more subsequent correct solutions than an urban mental walk after the incubation phase. This would help determine the time points at which this intervention could be most beneficial and provide its largest effects on creativity.

Another direction for future studies is to determine how long these effects of nature exposure may last. It is unclear whether the effect may be short-lived or last for an extended period. If it is determined that the effects of nature exposure last a short amount of time, this could also give more credence to the argument of only receiving nature exposure during the incubation phase, since it would be crucial to receive the exposure at a critical time point, rather than prior to being presented a problem. If this is true, this could also partially explain why no differences were found in our current AUT scores, as the AUT was presented after the RAT, meaning more time had passed since exposure to the mental walk. Future work can also determine whether the same guided imagery audio files produce the same level of immersion over time. It could be that with multiple exposures, one will learn to proceed through the mental walk with more ease and efficiency, resulting in enhanced effects. This could also lead to less of a mental burden to create the mental imagery involved. In return, there could be an enhanced improvement in creativity. However, it could also be the case that as the same guided imagery audio file is presented multiple times, the effect may wear off and it may not be as salient or effective with each subsequent mental walk. Thus, future studies need to determine whether the same mental imagery experiences have consistent effects over time. Future studies should also attempt to utilize this guided imagery approach in a laboratory setting to provide the highest likelihood that participants are focused on the task and have no distractions to disrupt their immersion.

Lastly, our findings may be relevant to existing literature examining the impact of stress on creativity. A meta-analysis found the relationship between stress and creativity is context-dependent, such that certain levels and types of stressors can increase creativity, whereas others can decrease creativity [81]. In addition, one study found that stress has a negative impact on divergent thinking, but not convergent thinking [82]. There are also numerous studies showing that nature exposure, both real and simulated, can reduce stress levels [83, 84]. Thus, an important future direction concerns determining whether nature exposure’s impact on different types of creativity may be moderated or mediated by stress reduction.

## Conclusion

While many studies have examined the impact of nature on creativity, none have examined the impact of internally experienced nature through guided imagery. The current study provides preliminary evidence that taking a mental walk in nature through the use of guided imagery audio files can improve performance on convergent thinking (though the performance improvements did not differ from a mental walk in urban environments). The same effect was not found for the urban mental walk, showing that the environment of the guided imagery experience plays an important role in convergent thinking. This suggests that certain people who are not able to easily access nature due to physical impairments, climate, or geographical limitations, can still potentially receive the creative benefits of nature exposure without needing to be physically immersed in it. These findings add to the growing field of research that shows that nature is beneficial to us in many ways and adds further evidence to the robustness of this effect to exist across such a vast range of modalities.

## Supporting information

S1 File Supplemental materials and analyses. (DOCX)
