# Supplementary material for: The effect of nature on creativity through mental imagery
Source: PLoS One. 2025 Jan 9;20(1):e0315141. doi: 10.1371/journal.pone.0315141 (PMC11717296; doi:10.1371/journal.pone.0315141)
Supplement: S1 File — (DOCX) [file pone.0315141.s001.docx]

**The Effect of Nature on Creativity Through Mental Imagery**

**S1 Supporting Information**

Aaron C. Drake, Fiza Hasan, Arianna Gibson, Julia W. Y. Kam

**Guided Imagery Scripts**

**Nature:**

0:00

“Begin by finding a comfortable position, either sitting or lying down in a location where you will not be interrupted. You are about to go through a guided imagery exercise. As you are listening, imagine yourself in the described situation and fully experience the situation you are imagining. To start, close your eyes. Imagine yourself walking on a path through a forest. The path is soft beneath your shoes, there is a mixture of soil, fallen leaves, pine needles, and moss. Continue walking down this path.

1:00 
Take a moment to look at your surroundings in this forest. Notice what you see in front of you. Notice the colours. The colours may be rich and deep, or light and faded. Notice the shapes and outlines in your environment. The shapes may be curved, linear, crooked or straight. Take a moment to notice the details in your surroundings, and how you are experiencing this moment.

2:00

The air is warm and comfortable. Sun filters through the trees, making a moving dappled pattern on the ground before you. Listen to the sounds of the forest... These sounds may be close by or somewhere far away. Take a moment to notice the details in your surroundings, and how you are experiencing this moment.

3:00

As you walk through the forest, you feel your arms swing in rhythm with your walking. Admire the scenery around you. Notice the movements in the environment. Focus on one of the movements and the way in which it moves. Notice the speed at which it moves. It may be moving fast, slow or at an even pace. It may have a rhythm. Notice the direction it moves. It may be moving in the same direction or back and forth or constantly changing direction. Take a moment to notice the details in your surroundings, and how you are experiencing this moment.

4:30 
As you continue to walk through the forest, you begin to climb up a slight incline. You easily tread along the woodchips on the path. You also notice the small trees grow at the sides of the path. The breeze continues to blow through the treetops, with fallen leaves being blown around by the wind.

5:00

Lean your head back and look up. You see tall trees grow on either side of the path. Picture the variety of trees around you. Some have smooth, white bark. Others are darker, with coarse, heavy bark, deeply grooved. Enjoy the colors of the bark on the trees – they may be white, brown, black. You admire the rough, brown bark of pine trees and enjoy the fresh pine scent.  Take a moment to notice the details in your surroundings, and how you are experiencing this moment.

6:00

As you continue walking through the forest, you can see a creek faintly in the distance. The air around you is fresh, and filled with the scent of trees, soil, and streams. Notice the smells. Is it pleasant or unpleasant, sharp or subtle? Take a moment to notice the details in your surroundings, and how you are experiencing this moment.

7:00 
As you continue walking, you get closer to a running stream.  The path curves up ahead. You can see sunlight peeking through a canopy of trees, casting golden rays on the ground around you.

7:30 
As you round the corner, you see a clearing in the trees up ahead. You are growing tired from your journey.

8:00 
Imagine yourself walking toward the clearing and the stream. Up ahead is a large, smooth rock... like a chair waiting for you to rest. The rock is placed perfectly near the edge of the stream.   
Sit or lie down on the rock if you wish. You feel very comfortable and at ease. The sun shines down on you. Notice the sensations on your skin. Reach your hand out and touch something around you. Notice how it feels against your skin as you are touching it. It may be rough, smooth, warm, cool, soft, prickly. Take a moment to notice the details in your surroundings, and how you are experiencing this moment.

9:00 
Feel the sun warming your body as you sit on the rock. Your body becomes very warm, and very heavy. Enjoy the landscape around you.

9:30 
Now take a moment to stop. You are about to leave this place. Take a moment to acknowledge the surrounding environment and the experience you have had.  Now slowly bring yourself back to the room. When you are ready, gently open your eyes and return to full wakefulness. You have completed the session for the guided imagery exercise. Please continue with the rest of the study.”

**Urban:**

0:00

“Begin by finding a comfortable position, either sitting or lying down in a location where you will not be interrupted. You are about to go through a guided imagery exercise. As you are listening, imagine yourself in the described situation and fully experience the situation you are imagining. To start, close your eyes.

0:30

Imagine yourself walking on a sidewalk through a city. The white concrete is firm and stable beneath your shoes. Continue walking down this sidewalk.

1:00

Take a moment to look at your surroundings in this city. Notice what you see in front of you. Notice the colours. The colours may be rich and deep, or light and faded. Notice the shapes and outlines in your environment. The shapes may be curved, linear, crooked or straight. Take a moment to notice the details in your surroundings, and how you are experiencing this moment.

2:00

The air is warm and comfortable. The sun shines around the tall buildings, making a steady pattern of shading on the ground before you. Listen to the sounds of the city. These sounds may be close by or somewhere far away. Take a moment to notice the details in your surroundings, and how you are experiencing this moment.

3:00

As you walk through the city, you feel your arms swing in rhythm with your walking.

Admire the scenery around you. Notice the movements in the environment. Focus on one of the movements and the way in which it moves. Notice the speed at which it moves. It may be moving fast, slow or at an even pace. It may have a rhythm. Notice the direction it moves. It may be moving in the same direction or back and forth or constantly changing direction. Take a moment to notice the details in your surroundings, and how you are experiencing this moment.

4:30

As you continue to walk through the city, you begin to climb up a slight incline. You easily tread along the smooth concrete on your path. You also notice the small shops on both sides of the path. The breeze continues to blow through the buildings, with empty cans and flyers being blown around by the wind.

5:00

Lean your head back and look up. You see tall skyscrapers on the other side of the street. Picture the variety of buildings around you. Some have smooth, dark windows. Others are lighter, with coarse, deeply grooved concrete. Enjoy the look of the different buildings – the many combinations of color and texture. Take a moment to notice the details in your surroundings, and how you are experiencing this moment.

6:00

As you continue walking through the city, you can see a café faintly in the distance. The air around you is fresh, and filled with all kinds of smells on the street.  Notice the smells. Is it pleasant or unpleasant, sharp or subtle? Take a moment to notice the details in your surroundings, and how you are experiencing this moment.

7:00

As you continue walking, you get closer to the cafe. The path curves up ahead. You can see sunlight streaming onto the sidewalk.

 7:30

As you take a slight left, you finally approach the café and see an open table outside. You are growing tired from your journey.

8:00

Imagine yourself walking toward the open table of the cafe. The chair seems to be waiting just for you. The chair is placed perfectly so that the sun will not shine in your eyes. Sit down in the chair if you wish. You feel very comfortable and at ease. The sun shines down on you. Notice the sensations on your skin. Reach your hand out and touch something around you. Notice how it feels against your skin as you are touching it. It may be rough, smooth, warm, cool, soft, prickly. Notice the textures.  Take a moment to notice the details in your surroundings, and how you are experiencing this moment.

9:00

Feel the sun warming your body as you sit in the chair. Your body becomes very warm, and very heavy. Enjoy the landscape around you.

9:30

Now take a moment to stop. You are about to leave this place. Take a moment to acknowledge the surrounding environment and the experience you have had.  Now slowly bring yourself back to the room. When you are ready, gently open your eyes and return to full wakefulness. You have completed the session for the guided imagery exercise. Please continue with the rest of the study.”

**Remote Associates Test (RAT) Versions and Items**

**RAT Practice Items:**

Flag, Vault, Fishing = Pole

Food, Front, Drug = Store

Due, Life, Tense = Past

**RAT Version A:**

Night, Wrist, Stop = Watch

Fountain, Baking, Can = Soda

Cracker, Fly, Fighter = Fire

Safety, Cushion, Point = Pin

Political, Surprise, House = Party

Worm, Shelf, End = Book

Print, Berry, Bird = Blue

Cadet, Capsule, Ship = Space

Fox, Man, Peep = Hole

Peach, Arm, Tar = Pit

Dust, Cereal, Fish = Bowl

Home, Sea, Bed = Sick

French, Car, Shoe = Horn

Main, Sweeper, Light = Street

Work, Clip, Wall = Paper

Dress, Dial, Flower = Sun

Eight, Skate, Stick = Figure

House, Ever, Pepper = Green

Foul, Ground, Mate = Play

Tail, Water, Flood = Gate

**RAT Version B:**

Show, Life, Row = Boat

Dew, Comb, Bee = Honey

Flake, Mobile, Cone = Snow

Cane, Rush, Plum = Sugar

Measure, Worm, Video = Tape

Piece, Mind, Dating = Game

Pie, Luck, Belly = Pot

Stick, Maker, Point = Match

Water, Mine, Shaker = Salt

Palm, Top, House = Tree

Wheel, Hand, Shopping = Cart

Nuclear, Feud, Album = Family

Cross, Rain, Tie = Bow

Station, Mask, Natural = Gas

Age, Mile, Sand = Stone

Health, Taker, Less = Care

Force, Line, Head = Air

Master, Toss, Finger = Ring

Hammer, Gear, Hunter = Head

Cover, Arm, Wear = Under

**RAT Version C:**

Loser, Throat, Spot = Sore

Duck, Fold, Dollar = Bill

Aid, Rubber, Wagon = Band

Dream, Break, Light = Day

High, Book, Home = School

River, Note, Account = Bank

Opera, Hand, Dish = Soap

Fur, Rack, Tail = Coat

Sleeping, Bean, Trash = Bag

Light, Birthday, Stick = Candle

Right, Cat, Carbon = Copy

Sandwich, House, Golf = Club

Pike, Coat, Signal = Turn

Boot, Summer, Ground = Camp

Wagon, Break, Radio = Station

Lift, Card, Mask = Face

Guy, Rain, Down = Fall

Animal, Back, Rat = Pack

Way, Board, Sleep = Walk

Point, List, Mate = Check

**RAT Version D:**

Cream, Skate, Water = Ice

Rocking, Wheel, High = Chair

Preserve, Ranger, Tropical = Forest

Fish, Mine, Rush = Gold

Sense, Courtesy, Place = Common

Flower, Friend, School = Girl

Date, Alley, Fold = Blind

Hound, Pressure, Shot = Blood

Shine, Beam, Struck = Moon

Food, Forward, Break = Fast

Basket, Eight, Snow = Ball

Wood, Paint, Hair = Brush

Mill, Tooth, Dust = Saw

Office, Mail, Lock = Box

Notch, Hill, Secret = Top

Catcher, Food, Hot = Dog

Officer, Cash, Larceny = Petty

Down, Question, Check = Mark

Carpet, Alert, Ink = Red

Change, Circuit, Cake = Short

**Alternate Uses Test (AUT) Versions and Items**

**AUT Version A:**

Brick

Barrel

Fishing Net

**AUT Version B:**

Car Tire

Tin Can

Spoon

**AUT Version C:**

Paperclip

Knife

Washcloth

**AUT Version D:**

Pencil

Clothes Hanger

Broom

**Imagination Manipulation Check Items**

**Please rate the level of detail present in your previously imagined mental walk in the following different aspects.**

1. ***Visual aspects of your mental walk***

1 = None at all, 2 = A little, 3 = Some, 4 = Quite a lot, 5 = Very much

1. ***Auditory/sound aspects of your mental walk***

1 = None at all, 2 = A little, 3 = Some, 4 = Quite a lot, 5 = Very much

1. ***Spatial arrangement of environmental objects of your mental walk***

1 = None at all, 2 = A little, 3 = Some, 4 = Quite a lot, 5 = Very much

1. ***Did you have a specific location in mind while imagining yourself taking this walk?***

o Yes. Please state specific location:_________________
o No

1. ***How often did you stay in the first-person perspective during the imagery?***

1 = Not at all, 2 = Rarely, 3 = Some of the time, 4 = Most of the time, 5 = The whole time

1. ***Please rate how often your mind wandered away from the guided mental imagery during the task.***

1 = Not at all, 2 = Rarely, 3 = Some of the time, 4 = Most of the time, 5 = The whole time

1. ***Please rate how mentally fatigued you feel right now.***

1 = Not at all, 2 = A little, 3 = Some, 4 = Quite a lot, 5 = Very much

1. ***Please rate how positive you feel right now.***

1 = Not at all, 2 = A little, 3 = Some, 4 = Quite a lot, 5 = Very much

1. ***Please rate how negative you feel right now.***

1 = Not at all, 2 = A little, 3 = Some, 4 = Quite a lot, 5 = Very much

**Pre-Registered Analyses**

**Control Analyses**

We implemented four control analyses. First, we checked for any ordering effects for the order in which participants received the mental walk condition (i.e. natural walk first and urban walk first). To examine this, we implemented a repeated-measures ANCOVA that included the pre-walk RAT and AUT scores as covariates, the condition they received first as a between-subjects variable, the condition of the walk as a within-subjects variable, and their post-walk RAT and AUT scores as the dependent variables. The results showed no significant differences between those who received the natural environment condition first and those who received the urban environment condition first for the RAT scores, *F*(1, 93) = 0.51, *p* = .477, η_p_^2^ = .01; originality scores of the AUT, *F*(1, 93) = 0.71, *p* = .401, η_p_^2^ = .01; or fluency scores of the AUT, *F*(1, 93) = 0.51, *p* = .478, η_p_^2^ = .01. Therefore, all participants were included in all other analyses regardless of which condition they first completed.

Our second set of control analyses examined whether a person’s ability to create mental imagery on a regular basis may impact the effectiveness of the mental walk condition on their creativity scores. Participants were split into high and low imaginative groups using a median split based on their scores on the Complexity factor of the Four Factor Imagination Scale. This resulted in 34 participants being labeled as low imaginative, and 47 as high imaginative. Those who fell on the median split score of 4 were excluded from this analysis (*n* = 16). We then implemented the same model of ANCOVA as above without the ordering of conditions variable, and instead included a group variable (low and high imaginative abilities) as a between-subject factor. There was no significant effect of imaginative abilities on post-walk RAT scores, with low imaginative (*M* = 7.99 [7.31, 8.68], *SE* = 0.35) and high imaginative participants (*M* = 8.06 [7.48, 8.64], *SE* = 0.29) scoring similarly on both post-walk RAT scores; *F*(1, 76) = 0.02, *p* = .890, η_p_^2^ = .00. For post-walk originality scores on the AUT, there was no difference between low imaginative (*M* = 1.36 [1.31, 1.41], *SE* = 0.03) and high imaginative (*M* = 1.35 [1.31, 1.39], *SE* = 0.02) participants either; *F*(1, 76) = 0.07, *p* = .793, η_p_^2^ = .00. The same was found for post-walk fluency scores on the AUT, with low imaginative (*M* = 16.80 [15.64, 17.96], *SE* = 0.58) and high imaginative (*M* = 16.47 [15.49, 17.46], *SE* = 0.49) participants scoring similarly; *F*(1, 76) = 0.18, *p* = .671, η_p_^2^ = .00. Since it appears that imaginative abilities did not impact creativity scores, it would be unlikely that accounting for this measure would impact the results. However, for the sake of completeness, we implemented analyses in accordance with our pre-registration utilizing imaginative abilities as a covariate, and report these results in the Covariate Analyses section below. These results suggest that one’s ability to create mental imagery as captured by the Four Factor Imagination Scale did not modulate their performance on either convergent or divergent creativity following a mental walk in either environment.

In our third set of control analyses, we examined whether one’s perception of how restorative their mental walk was may account for variance in their performance on the RAT and AUT scores. To test this, we performed two correlation analyses between their Perceived Restorativeness Scale scores and post-walk creativity scores separately for both natural and urban environments. There was no significant correlation between their Perceived Restorativeness Scale scores and post-walk RAT scores for the natural environment (*r* = –.06 [-.26, .14], *p* = .534) and urban environment (*r* = .18 [-.02, .37], *p* = .080). There was also no significant correlation between their Perceived Restorativeness Scale scores and post-walk originality AUT scores for the natural (*r* = –.01 [-.21, .19], *p* = .953) and urban environments (*r* = –.11 [-.30, .09], *p* = .289). The same was found for post-walk fluency AUT scores in the natural environment (*r* = .04 [-.16, .24], *p* = .691). For the urban environment, (*r* = .20 [.00, .38], *p* = .052), individuals who perceived urban environments to be more restorative provided numerically more responses on the AUT; however, this correlation only approached significance. Together, these results suggest that one’s beliefs about nature’s (or urban setting’s) restoration capacity did not relate to their performance on the creativity tasks.

Finally, to test whether general beliefs of environments and their benefit to creativity may impact the effect of the mental walk, we performed independent-samples t-tests to compare those who believed that natural or urban environments are generally beneficial for creativity to those who did not. For the nature condition, given the large imbalance between the two groups (only 5 participants responded “No”, while 92 responded “Yes”), we did not implement this planned analysis. For the urban condition, the responses were slightly more balanced (21 responded “No” and 76 responded “Yes”). Therefore, we examined the relationship between beliefs about urban environments being beneficial to creativity (yes vs. no), and their post-urban walk creativity scores. Those who responded “Yes” (*M* = 8.21, *SD* = 4.22) did not score significantly different on post-walk RAT scores compared to those who responded “No” (*M* = 6.86, *SD* = 4.32); *∆M* = 1.35 [-0.72, 3.43], *t*(95) = 1.30, *p* = .198, *d* = 0.32. Those who responded “Yes” (*M* = 1.34, *SD* = 0.19) did not score significantly different on the post-walk originality scores compared to those who responded “No” either (*M* = 1.38, *SD* = 0.24); *∆M* = -0.03 [-0.15, 0.08], *t*(27.27) = 0.58, *p* = .569, *d* = 0.16. The same was found between “Yes” (*M* = 16.14, *SD* = 6.73) and “No” (*M* = 15.71, *SD* = 6.55) groups for the post-walk fluency scores as well; *∆M* = 0.43 [-2.85, 3.71], *t*(95) = 0.26, *p* = .795, *d* = 0.06. These results indicate that one’s general beliefs about the benefits of an urban environment on creativity had no impact on their performance on the creativity tasks. Importantly, an examination of participants’ responses to our control questions revealed that they were engaged in the imagination and mind wandered away from their mental walk on average some of the time. Responses to the imagination manipulation checks and mind wandering questions are reported in Supplementary Table 1 and 2.

**Covariate Analyses**

To examine whether there would be conditional differences while controlling for connection to nature (as indexed by the Nature Relatedness Scale - Experience Subscale) and their imagination abilities (as indexed by the complexity factor of the Four Factor Imagination Scale), we included these two additional variables as covariates and the same ANCOVA as the main analysis was performed. There was not a significant difference between the nature (*M* = 8.40 [7.87, 8.93], *SE* = 0.27) and urban (*M* = 7.92 [7.37, 8.47], *SE* = 0.28) conditions for the RAT scores, *F*(1, 92) = 0.28, *p* = .600, η_p_^2^ = .00. We then also included the Nature Relatedness Scale - Experience Subscale and the complexity factor of the Four Factor Imagination Scale as covariates for both AUT measures as the main analyses. There were still no significant differences found between the nature (*M* = 1.35 [1.32, 1.39], *SE* = 0.02) and urban (*M* = 1.35 [1.31, 1.39], *SE* = 0.02) conditions for originality, *F*(1, 92) = 2.00, *p* = .162, η_p_^2^ = .02). There were also no significant differences between the nature (*M* = 16.94 [16.08, 17.79], *SE* = 0.43) and urban (*M* = 16.05 [15.26, 16.84], *SE* = 0.40) conditions for fluency, *F*(1, 92) = 1.24, *p* = .268, η_p_^2^ = .01.

| **Supplementary Table 1**  *Descriptive Statistics for Imagination Manipulation Check Items* | | |
| --- | --- | --- |
|  | **Environment** | |
| **Item** | **Natural** | **Urban** |
| Visual aspects | *M* = 4.06 (*SD* = 0.79) | *M* = 3.92 (*SD* = 0.83) |
| Auditory aspects | *M* = 4.40 (*SD* = 0.84) | *M* = 4.28 (*SD* = 0.69) |
| Spatial arrangement | *M* = 3.70 (*SD* = 1.00) | *M* = 3.64 (*SD* = 0.83) |
| First-person | *M* = 3.99 (*SD* = 1.03) | *M* = 4.09 (*SD* = 0.90) |
| Mind wandered | *M* = 2.73 (*SD* = 0.69) | *M* = 2.62 (*SD* = 0.76) |
| Mental fatigue | *M* = 2.55 (*SD* = 1.05) | *M* = 2.70 (*SD* = 1.11) |
| Positive feeling | *M* = 3.61 (*SD* = 0.90) | *M* = 3.41 (*SD* = 0.99) |
| Negative feeling | *M* = 1.55 (*SD* = 0.82) | *M* = 1.85 (*SD* = 0.97) |
| Specific location | “Yes” = 54.6% | “Yes” = 75.3% |

Note: All of the questions above except for the last one were rated on a scale of 1 to 5.

| **Supplementary Table 2**  *Frequency of Responses for Post Audio Mind Wandering Question* | | |
| --- | --- | --- |
|  | **Environment** | |
| **Response Value** | **Natural** | **Urban** |
| 1 (Not at all) | *n* = 3 | *n* = 4 |
| 2 (Rarely) | *n* = 30 | *n* = 41 |
| 3 (Some of the time) | *n* = 54 | *n* = 41 |
| 4 (Most of the time) | *n* = 10 | *n* = 13 |
| 5 (The whole time) | *n* = 2 | *n* = 0 |

Note: Participants who reported mind wandering the whole time (response = 5) during the audio were excluded from the main analyses as reported in the main manuscript.

**Tests of Assumptions**

To fully check all assumptions for our analyses, normality tests were performed first. Results from these checks are reported in Supplementary Table 3. We also visually inspected all Q-Q and box plots and noted no significant outliers in any of the variables. We then checked to determine whether there was a relationship between the covariate (pre-walk scores) and the dependent variable (post-walk scores) for each analysis. Pre-nature walk RAT scores positively correlated with post-nature walk RAT scores (*r* = .75, *p* <.001), and pre-urban walk RAT scores positively correlated with post-urban walk RAT scores (*r* = .76, *p* <.001). Pre-nature walk AUT originality scores were not correlated with post-nature walk AUT originality scores (*r* = .10, *p* = .338), while the pre-urban walk AUT originality scores positively correlated with the post-urban walk AUT originality scores (*r* = .31, *p* = .002). The pre-nature walk AUT fluency scores positively correlated with the post-nature walk AUT fluency scores (*r* = .82, *p* < .001), and the pre-urban walk AUT fluency scores positively correlated with the post-urban walk AUT fluency scores (*r* = .77, *p* < .001). Thus, all but one covariate and dependent variable relationship was significantly correlated. The assumption of sphericity is met for all analyses given that there were only two levels of the independent variable (nature vs. urban). Thus, all Mauchly’s tests of sphericity result in a perfect value of 1.00.

| **Supplementary Table 3**  *Tests of Normality* | | | |
| --- | --- | --- | --- |
| **Measure** | **Skewness** | **Kurtosis** | **Shapiro-Wilk** |
| Pre-nature RAT | 0.51 (*SE* = 0.25) | 0.26 (*SE* = 0.49) | 0.97 (*p* = .049) |
| Post-nature RAT | 0.28 (*SE* = 0.25) | 0.32 (*SE* = 0.49) | 0.97 (*p* = .041) |
| Pre-urban RAT | 0.55 (*SE* = 0.25) | 0.43 (*SE* = 0.49) | 0.97 (*p* = .025) |
| Post-urban RAT | 0.34 (*SE* = 0.25) | -0.38 (*SE* = 0.49) | 0.97 (*p* = .014) |
| Pre-nature AUT Originality | 0.33 (*SE* = 0.25) | -0.36 (*SE* = 0.49) | 0.98 (*p* = .280) |
| Post-nature AUT Originality | 0.09 (*SE* = 0.25) | -0.52 (*SE* = 0.49) | 0.99 (*p* = .370) |
| Pre-urban AUT Originality | 0.36 (*SE* = 0.25) | -0.76 (*SE* = 0.49) | 0.96 (*p* = .007) |
| Post-urban AUT Originality | 0.64 (*SE* = 0.25) | 0.17 (*SE* = 0.49) | 0.97 (*p* = .011) |
| Pre-nature AUT Fluency | 0.40 (*SE* = 0.25) | 0.60 (*SE* = 0.49) | 0.99 (*p* = .337) |
| Post-nature AUT Fluency | 0.81 (*SE* = 0.25) | 0.95 (*SE* = 0.49) | 0.96 (*p* = .003) |
| Pre-urban AUT Fluency | 0.48 (*SE* = 0.25) | -0.04 (*SE* = 0.49) | 0.97 (*p* = .053) |
| Post-urban AUT Fluency | 0.58 (*SE* = 0.25) | 1.04 (*SE* = 0.49) | 0.98 (*p* = .069) |

**Exploratory Analyses**

Recent work has implemented AI-powered semantic processing methods to score responses on the AUT through large language models (Organisciak et al., 2023). This method attempts to overcome reliability issues with human scoring methods, as well as making the scoring much faster and cost-effective. These scores are given on a scale of 1-5, with 5 being the most original. We utilized this AI-powered scoring method (using the “ocsai-chatgpt” model) to re-score the originality aspect of the AUT responses to determine if this led to changes in our results. We implemented the identical statistical analyses reported in the manuscript, with the only difference being that the AI-generated originality scores were used instead of the raters’ scores. To examine whether each mental walk environment condition improved participants’ AUT originality scores from pre- to post-walk, we conducted paired-samples *t*-tests for originality. For the nature environment, there were no significant differences from pre-walk (*M* = 2.47, *SD* = 0.36) to post-walk scores (*M* = 2.47, *SD* = 0.35); *∆M* = 0.01 [-0.13, 0.12], *t*(96) = 0.12, *p* = .902, *d* = 0.01). The same was true for the urban environment from pre-walk (*M* = 2.51, *SD* = 0.34) to post-walk scores (*M* = 2.48, *SD* = 0.36); *∆M* = 0.04 [-0.08, 0.15], *t*(96) = 0.62, *p* = .539, *d* = 0.06.

To further examine whether the two environment conditions (natural and urban) differentially impacted AI-generated originality scores at the post-walk timepoint while controlling for their pre-walk scores, a repeated-measures ANCOVA was performed that included the pre-walk scores as a covariate, the mental walk condition as the within-subjects variable, and post-walk scores as the dependent variable. Similar to the raters’ scores, this new scoring method resulted in non-significant differences between the post-nature (*M* = 2.47 [2.43, 2.52], *SE* = 0.02) and post-urban walk (*M* = 2.48 [2.43, 2.53], *SE* = 0.03) conditions for originality (*F*(1, 94) = 0.05, *p* = .823, η_p_^2^ = .00). We then performed the same analysis including the NRS and the FFIS as covariates as mentioned above. There were no significant differences found between the nature (*M* = 2.47 [2.43, 2.52], *SE* = 0.02) and urban (*M* = 2.48 [2.43, 2.53], *SE* = 0.03) conditions for AI-scored originality, *F*(1, 92) = 0.10, *p* = .757, η_p_^2^ = .00).

**References**

Organisciak, P., Acar, S., Dumas, D., & Berthiaume, K. (2023). Beyond semantic distance: Automated scoring of divergent thinking greatly improves with large language models. *Thinking Skills and Creativity*, *49*, 101356. https://doi.org/10.1016/j.tsc.2023.101356
